# From microbial promotion to co-exposure governance: policy implications of *Candida albicans* in oral carcinogenesis

**DOI:** 10.1080/20002297.2026.2731658

**Published:** 2026-09-10

**Authors:** Ardiyansah, Fitrotun Niswah

**Affiliations:** a Department of Public Administration, Faculty of Social and Political Sciences, Universitas Negeri Surabaya, Surabaya, Indonesia

To the Editor,

We read with great interest the study by Wang et al. [1], which asks whether *Candida albicans* is an inducer or, more plausibly, a promoter of oral carcinogenesis. The distinction is more than semantic. In their long-term murine model, *C. albicans* alone did not produce oral cancer, yet exposure increased epithelial proliferation, altered cancer-related gene expression, and generated signals consistent with DNA damage. When oral mucosa had first been exposed to the tobacco-mimetic carcinogen 4-nitroquinoline-1-oxide (4NQO), *C. albicans* accelerated tumour formation and amplified inflammatory and immune-response pathways. The small chronic hyperplastic candidiasis cohort also showed a mutational pattern that the authors interpreted as compatible with co-contribution from *Candida* and smoking. This supports a promoter, rather than stand-alone initiator, interpretation.

This interpretation has gained human support since the publication of Wang et al. In a multicenter prospective study in the same journal, Jiang et al. followed 734 patients with oral potentially malignant disorders and found malignant transformation in 6.8%; high *C. albicans* burden was independently associated with transformation (adjusted HR 2.84, 95% CI 1.40−5.75), with the strongest association reported in leukoplakia [2]. A 2026 Journal of Oral Microbiology cohort likewise found higher relative abundance of *C. albicans* and a greater prevalence of a *Candida*-dominated salivary mycotype in patients with oral squamous cell carcinoma than in non-cancer controls [3]. Together with experimental evidence implicating the IL-17A/IL-17RA-macrophage axis [4], the emerging picture is not of *Candida* as a universal carcinogen, but as a context-dependent biological modifier. This context-dependent interpretation argues against both dismissal of the evidence and population-wide fungal screening or prophylactic antifungal treatment.

We suggest that the translational novelty lies in what may be termed co-exposure governance. Public health systems commonly place tobacco control, oral fungal disease, potentially malignant oral lesions, and cancer referral in separate administrative pathways. Yet the biology described by Wang et al. implies that risk may arise precisely at their intersection. From a public administration perspective, the relevant unit of action is therefore not *C. albicans* alone, but the pathway through which microbial burden, tobacco exposure, lesion phenotype, clinical follow-up, and referral capacity combine. This reframing moves the discussion from a single-pathogen question toward integrated risk management, with clear implications for service design, accountability, surveillance, and continuity of care.

Indonesia provides a particularly relevant setting in which to test this approach. The Global Adult Tobacco Survey reported tobacco use in 34.5% of Indonesian adults and 65.5% of men [5]. GLOBOCAN 2022 estimated 6,515 new cancers of the lip and oral cavity and 3,546 deaths in Indonesia [6]. At the same time, a recent review of the Indonesian oral health system identified geographic barriers, uneven distribution of dental personnel, limited prevention and promotion, and weaknesses in monitoring and evaluation [7]. Government Regulation No. 28 of 2024 has strengthened tobacco-control measures, including raising the purchasing age to 21 years and banning single-stick sales, creating a timely policy window for linking tobacco prevention with oral-health risk management [8]. These national conditions do not establish the prevalence or causal role of *C. albicans* in Indonesian oral cancer, but they make the interaction hypothesis highly relevant for implementation research.

A cautious policy response would therefore focus on targeted integration rather than mass testing. In primary-care and dental settings, especially for patients with oral potentially malignant disorders or persistent or recurrent candidiasis, a practical risk bundle could combine structured tobacco history, lesion documentation, *Candida* assessment when clinically indicated, cessation support, defined referral criteria, and recall tracking. The WHO South-East Asia oral health action plan already calls for integration of oral health with primary health care and noncommunicable disease programmes, stronger governance, surveillance, and a 33.3% relative reduction in premature oral-cancer mortality by 2030 [9]. The next step should be a prospective Indonesian implementation study asking whether adding fungal burden to established clinical and tobacco-risk variables improves risk stratification, referral completion, time to specialist assessment, stage at diagnosis, and cost-effectiveness. Such a study should also examine equity across urban, rural, and underserved populations.

Wang et al. shift the scientific question from whether *C. albicans* independently causes oral cancer to when it meaningfully amplifies malignant risk. We believe the corresponding policy question is how health systems can recognise and manage that interaction without medicalizing colonisation or expanding antifungal use beyond evidence. For Indonesia and comparable high-tobacco settings, the most defensible near-term innovation is not a new stand-alone fungal programme, but an integrated co-exposure pathway that helps high-risk patients avoid being lost between dental care, tobacco cessation, pathology, and cancer services. This is a testable, human-centred translation of the biological evidence and a practical agenda for both oral microbiology and public administration.
